# Short-latency afferent inhibition and somato-sensory evoked potentials during the migraine cycle: surrogate markers of a cycling cholinergic thalamo-cortical drive?

**DOI:** 10.1186/s10194-020-01104-7

**Published:** 2020-04-16

**Authors:** Gianluca Coppola, Davide Di Lenola, Chiara Abagnale, Fabio Ferrandes, Gabriele Sebastianelli, Francesco Casillo, Cherubino Di Lorenzo, Mariano Serrao, Maurizio Evangelista, Jean Schoenen, Francesco Pierelli

**Affiliations:** 1 Department of Medico-Surgical Sciences and Biotechnologies, Sapienza University of Rome Polo Pontino, Corso della Repubblica 79, 04100 Latina, Italy; 2 Università Cattolica del Sacro Cuore/CIC, Istituto di Anestesiologia, Rianimazione e Terapia del Dolore, Largo Agostino Gemelli 8, 00168 Rome, Italy; 3 Headache Research Unit, University Department of Neurology CHR, Citadelle Hospital. University of Liège, Boulevard du Douzième de Ligne 1, 4000 Liège, Belgium; 4 IRCCS – Neuromed, via Atinense, 18, 86077 Pozzilli, IS Italy

**Keywords:** Motor cortex inhibition, Thalamo-cortical activation, Sensorimotor integration, GABA, Episodic migraine without aura

## Abstract

**Background:**

Short-latency afferent inhibition (SAI) consists of motor cortex inhibition induced by sensory afferents and depends on the excitatory effect of cholinergic thalamocortical projections on inhibitory GABAergic cortical networks. Given the electrophysiological evidence for thalamo-cortical dysrhythmia in migraine, we studied SAI in migraineurs during and between attacks and searched for correlations with somatosensory habituation, thalamocortical activation, and clinical features.

**Methods:**

SAI was obtained by conditioning the transcranial magnetic stimulation-induced motor evoked potential (MEP) with an electric stimulus on the median nerve at the wrist with random stimulus intervals corresponding to the latency of individual somatosensory evoked potentials (SSEP) N20 plus 2, 4, 6, or 8 ms. We recruited 30 migraine without aura patients, 16 between (MO), 14 during an attack (MI), and 16 healthy volunteers (HV). We calculated the slope of the linear regression between the unconditioned MEP amplitude and the 4-conditioned MEPs as a measure of SAI. We also measured SSEP amplitude habituation, and high-frequency oscillations (HFO) as an index of thalamo-cortical activation.

**Results:**

Compared to HV, SAI, SSEP habituation and early SSEP HFOs were significantly reduced in MO patients between attacks, but enhanced during an attack. There was a positive correlation between degree of SAI and amplitude of early HFOs in HV, but not in MO or MI.

**Conclusions:**

The migraine cycle-dependent variations of SAI and SSEP HFOs are further evidence that facilitatory thalamocortical activation (of GABAergic networks in the motor cortex for SAI), likely to be cholinergic, is reduced in migraine between attacks, but increased ictally.

## Background

A number of neurophysiological and neuroimaging studies have shown dysfunctions of both somatosensory and motor systems in the brain of migraine patients [1–8]. Recently, the integration between the two systems was also found to be altered in migraineurs, especially during an attack [9, 10]. Part of the evidence comes from a study of the phenomenon of short-latency afferent inhibition (SAI) by which a peripheral sensory afferent volley inhibits the homotopic muscle response obtained by stimulation of the motor cortex [11]. SAI is typically studied by conditioning a transcranial magnetic stimulus (TMS)-evoked motor potential (MEP) by a preceding electrical stimulus of a peripheral nerve, usually the median nerve at the wrist. The peripheral electrical stimulus inhibits the motor-evoked potential and the degree of this inhibition depends on the interval between the sensory and the motor stimuli (ISI). For SAI to occur, both the motor and somatosensory systems must be functionally intact. SAI depends on the excitatory effect exerted by cholinergic thalamocortical afferents on inhibitory GABAergic cortical networks [11]. Previous studies have shown that migraineurs are characterized by ictal and interictal dysfunctions of both thalamic and cortical somatosensory nodes [2, 12–14], and of the cortical synaptic plasticity partially depending on GABAergic mechanisms [15–17].

SAI was previously reported to be decreased during the pre-ictal and ictal phases of episodic migraine [10], but in this study ISIs were predetermined and equal in all subjects. In fact, the SAI protocol should preferentially adjust ISIs according to the individual latency of the N20 component of the somatosensory potential evoked by the peripheral stimulus that indicates the arrival of the afferent volley in cortical area S1 [18, 19].

In the present study we applied such a protocol in healthy volunteers and migraine patients during and between attacks. We recorded SAI at 4 different ISIs, determined on the basis of the SSEP N20 latency in each individual as well as somatosensory high-frequency oscillatory activity reflecting thalamocortical activation, in order to evaluate sensorimotor control and its relationship to the activity of thalamocortical afferents. We searched for correlations between the neurophysiological data and clinical migraine features.

## Subjects and methods

### Participants

In 2016 and 2017 we prospectively recruited 30 patients with migraine without aura at the headache clinic of the Sapienza University of Rome Polo Pontino in Latina (Italy). Of these patients, 16 were recorded during the pain-free period (MO), i.e. at a distance of at least 3 days from the last and the next migraine attack at the time of the recording session. The remaining 14 patients were recorded within the first 8 h of a spontaneous migraine attack, during the headache phase (MI) (see Table 1 for the clinical characteristics). Patients who had received prophylactic therapy in the preceding 3 months or had any other neurological or psychiatric disorder were excluded. For comparison, we recruited 16 healthy volunteers (HV) from the medical and nursing staffs with no personal or family history of migraine or another primary headache. HVs were randomly recorded between patients. All female participants were recorded at mid-cycle at an average of 18.8 (HV), 17.4 (MO) or 17.2 days (MI) after the 1st day of the last menstruation). Clinical information was collected from headache diaries the participants had filled in for at least 1 month before the recording session. The study was conducted in accordance with the Declaration of Helsinki and was approved by the Ethical Committee of the ‘Sapienza’ University of Rome. All individuals were naïve to the study procedure and provided written informed consent.

**Table 1:** Demographic and clinical features of healthy volunteers (HV) and migraine patients without aura between (MO) and during (MI) the attacks. Data are expressed as means ± SD. (*p* < 0.05 * MO vs. HV, ** MI vs. MO)

*Characteristics*	HV (n = 16)	MO (*n* = 16)	MI (*N* = 14)
Women (n)	12	14	10
Age (years)	27.5 ± 9.4	28.0 ± 7.5	32.2 ± 9.2
Duration of migraine history (years)		12.3 ± 6.8	19.9 ± 10.9 *
Attack frequency/month (n)		2.9 ± 2.5	2.9 ± 2.9
Attack duration (hours)		15.2 ± 15.5	22.1 ± 21.9
Severity of headache (n)		8.0 ± 1.2	7.4 ± 0.9
Days from the last migraine attack (n)		16.4 ± 15.2	

### Transcranial magnetic stimulation (TMS)

TMS was performed using a MagStim rapid device (MagstimRapid, The Magstim Company Ltd., Whitland, South West Wales, UK) connected to a figure-of-8 coil of which each loop had a 9 cm outer diameter. The coil was placed on the right side of scalp in an optimal position to elicit an electromyographic response in the first dorsal interosseous muscle of the left. The optimal site was labelled with a red dermographic pencil. The motor evoked potential was recorded using silver-chloride cups with the active electrode positioned on the muscle and the reference electrode on the metacarpophalangeal junction of the index finger. The resting motor threshold (RMT) was defined as the minimal intensity needed to evoke an electromyographic response of at least 50 μV with 50% probability in a fully relaxed muscle.

### Somatosensory evoked potentials

SSEPs were obtained by electrical stimulation of the left median nerve at the wrist, at an intensity of 1.2 times the motor threshold of the thumb. The active recording electrode was positioned at the C3’ position of the 10–20 international system (2 cm posterior to C3), with the reference electrode at Fz; the ground electrode was positioned over the left arm. The electrocortical signals were amplified with Digitimer D360™ pre-amplifiers, recorded by a CED™ power1401 device (CED Ltd., Cambridge, UK), and off-line analysed with the Signal™ software package version 4.10 (CED Ltd). While subjects were sitting relaxed with open eyes and fixed their attention on the movement of their thumb, 300 non-artefacted traces were acquired at a frequency of 4.4 Hz. On the grand-average trace we measured the latency of the parietal components N20 and P25 and their respective peak-to-peak amplitudes. Subsequently, the 300 traces were averaged in 3 blocks of 100 in order to study SSEP habituation. The peak-to-peak amplitude N20-P25 was measured in each block and habituation was calculated as the slope of the regression line along the amplitudes of the 3 sequential blocks.

### Somatosensory high-frequency oscillations (HFOs)

To assess thalamocortical activity, we extracted HFOs from the broad-band SSEP signal using the method published elsewhere [20]. In brief, we applied a digital band-pass filter (450–750 Hz, 51 coefficients) on the grand-average SSEP. From the filtered trace we extrapolated the amplitude of the maximum peak of the two oscillation bursts, i.e. the one that occurs before the N20 peak of the broad-band SSEP (early HFOs), and the one that appears after the N20 peak (late HFOs). By identifying source activity from multichannel scalp recordings and the effect of pharmacological agents, previous studies have determined that the early HFO burst reflects thalamic/thalamocortical activity while the late burst is generated by cortical activation [21, 22].

### Short-latency afferent inhibition (SAI)

The recordings were performed in the afternoon between 2 P.M. and 7 P.M. while participants were sitting on a comfortable armchair with eyes closed. The peripheral conditioning stimuli on the median nerve (200 μs duration) were applied to the left wrist with a bipolar electrode at an intensity of 1.2 times the motor threshold. The intensity of the TMS test stimulus over the right motor cortex was set at 120% of the resting motor threshold.

We studied SAI using the following protocol. The peripheral electrical stimulus preceded the cortical magnetic stimulus by interstimulus intervals (ISIs) of 2, 4, 6, or 8 msec set with respect to the latency of the SSEP N20 peak in each subject. The baseline MEP was obtained without the conditioning stimulus. For each participant, 40 acquisitions were performed, 5 for each condition (baseline, 2, 4, 6, 8 ms ISIs), randomly applied with a 5-s intertrial interval, and averaged off-line in 5 blocks. For each block, we measured the average peak-to-peak MEP amplitude whereafter we computed the slope of MEP amplitude regression line between the unconditioned and the 4 conditioned recordings as an overall measure of the SAI effect. Three investigators (DDL, CDL, and CA), not involved in patients’ recruitment, performed the recordings. All recordings were anonymized and analyzed blindly off-line by one investigator (F.F).

#### Statistical analysis

We used the Statistica for Windows (StatSoft Inc.) version 8.0, for all analyses. An a posteriori sample size calculation based upon a recently published study where SAI was assessed with a similar protocol [10] showed that 7 subjects per group (standardized effect size of 2.0428) are needed to disclose a significant difference between HV and migraine patients during the attack (power 0.90, alpha error 0.05).

Descriptive analysis showed that MEP peak-to-peak amplitudes of the SAI were not normally distributed. After log transformation, all data reached a normal distribution (Kolmogorov-Smirnov test).

A General Linear Model approach was used to analyze the “between-subjects factor” × “within-subjects factors” interaction effect. The between-subjects factor was the variable “group” (HV, MO, and MI); for SAI the within-subjects factor was “ISI” (baseline, 2 ms, 4 ms, 6 ms, and 8 ms) and for SSEP it was “block” (from the 1st to the 3rd block). Two models of repeated measures ANOVA (rm-ANOVA) followed by univariate ANOVAs were employed to investigate the interaction effect. Univariate results were analyzed only if Wilks’ Lambda multivariate significance criterion was achieved.

A regression analysis was used to disclose linear trends in MEP amplitude across ISIs (slope) and in SSEP N20-P25 amplitude across blocks in each group. For SSEP, HFOs, MEP and MEP/SSEP slopes we employed one-way ANOVAs with factor “group” (HV, MO, MI), using least significant difference test for post hoc analysis. *P* < 0.05 was considered as statistically significant.

Pearson’s correlation test was used to search for correlations between MEP/SSEP amplitude slopes, early/late HFOs and clinical variables such as duration of episodic migraine history, mean monthly attack frequency, mean monthly attack duration, number of days since the last migraine attack, severity of migraine headache on a 0–10 visual analogue scale.

## Results

The clinical features did not differ between the 2 groups of migraineurs, except for a significantly longer duration of disease in patients recorded during an attack (F_1,29_ = 5.833, *p* = 0.023, Table 1).

### Somato-sensory broad-band evoked potentials (SSEP) and high frequency oscillations (HFO)

N20 and P25 latencies, N20-P25 amplitudes in the grand-average of 300 responses or in the 3 blocks of 100 sequential responses were not different between groups (F_2,45_ < 2.5, *p* > 0.05, Table 2).

**Table 2:** Latencies and amplitudes of the various cortical SSEP components after median nerve stimulation (mean ± standard deviation)

	HV (*n* = 16)	MO (*n* = 16)	MI (*N* = 14)
N20 (ms)	19.0 ± 0.9	18.6 ± 0.7	18.9 ± 0.9
P25 (ms)	23.6 ± 1.8	23.2 ± 1.7	23.0 ± 2.1
N20-P25 (μV)	2.3 ± 0.9	2.2 ± 0.9	1.8 ± 1.0
1st N20-P25 (μV)	2.8 ± 1.1	2.1 ± 0.9	2.1 ± 1.1
2nd N20-P25 (μV)	2.4 ± 0.9	2.5 ± 0.9	1.9 ± 1.0
3rd N20-P25 (μV)	2.5 ± 0.9	2.5 ± 0.9	1.9 ± 0.9
Slope (block 1–3)	− 0.33 ± 0.44 *	0.49 ± 0.51	−0.14 ± 0.32 **
Early HFOs amplitude (μV)	0.058 ± 0.022 *	0.039 ± 0.019	0.051 ± 0.025
Late HFOs amplitude (μV)	0.061 ± 0.039	0.043 ± 0.019	0.070 ± 0.033

(*p* < 0.05 * MO vs. HV, ** MI vs. MO) *HV* Healthy volunteers, *MO* Migraine patients between attacks, *MI* Migraine patients during attacks

**Fig. 1:**
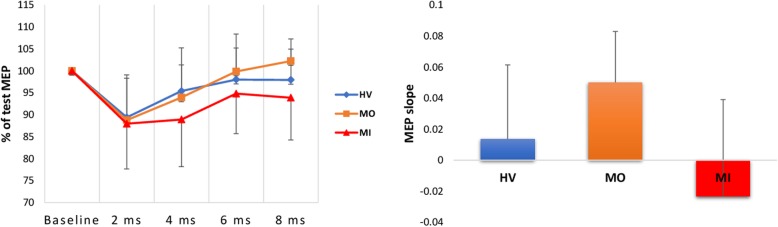
Left panel: Line plots of short-latency afferent inhibition (SAI) at the various interstimulus intervals in healthy volunteers (HV) and in migraine patients recorded between (MO) or during (MI) an attack. Right panel: Bar chart of the MEP slope (mean + sd) of the regression line between the unconditioned MEP amplitude and the 4-conditioned MEPs in the same subject groups. MEP = motor evoked potential

In the rm-ANOVA model with N20-P25 peak-to-peak amplitude as dependent variable, the multivariate test was significant for the “group” × “block” interaction (Wilks’ Lambda = 0.608, F_4,86_ = 7.354, *p* = 0.00004). In fact, in both HVs and patients during an attack, the linear regression slope of SSEP N20-P25 amplitudes over the 3 blocks showed a negative value, i.e. habituated (slope − 0.33 in HV and − 0.14 in MI, Table 2 & Fig. 1), while it was positive in patients between attacks, indicating a habituation deficit (+ 0.49, *p* < 0.001 MO vs. HV).

After applying the bandpass digital filter to the grand-average SSEP, amplitude of the early HFOs (F_2,45_ = 3.146, *p* = 0.048) was reduced in MO patients compared to HVs (*p* = 0.019), while it was within normal range in MI patients (*p* = 0.398). Amplitude of the late HFOs did not differ between groups (F_2,45_ = 2.848, *p* = 0.069, Table 2 & Fig. 1).

### Short-latency afferent inhibition (SAI)

There was no significant difference between groups in the motor thresholds after electrical stimulation of the median nerve or magnetic stimulation of the motor cortex (F_2,45_ = 1.40, *p* = 0.301; F_2,45_ = 1.30, *p* = 0.283 respectively).

In the rm-ANOVA model with MEP peak-to-peak amplitude as dependent variable, the multivariate test was significant for the “group” × “ISI” interaction (Wilks’ Lambda = 0.665, F_8,172_ = 2.04, *p* = 0.045). The linear regression slope of MEP amplitudes over the 5 conditioning latencies, indexing SAI, was significantly different between groups (F_2,45_ = 8.571, *p* = 0.001). The MEP amplitude slope showed a greater positive value in MO patients than in HVs (+ 242.3 in MO vs + 11.2 in HV, *p* = 0.039), while MI patients had a negative slope (− 129.6, *p* = 0.043 vs. HV) (Table 3). These results indicate that SAI is reduced between migraine attacks, but increased during an attack.

**Table 3:** Mean motor thresholds after median nerve and transcranial magnetic stimulation, baseline and conditioned amplitudes of motor evoked potentials (mean ± standard deviation) in healthy volunteers and migraine without aura patients between (MO) and during (MI) the attacks. (*p* < 0.05 * MO vs. HV, ** MI vs. MO)

	HV (*n* = 16)	MO (*n* = 16)	MI (*N* = 14)
Median nerve motor threshold (mA)	8.6 ± 1.8	7.4 ± 1.9	8.9 ± 2.7
Resting motor threshold (%)	58.3 ± 11.4	55.1 ± 6.0	60.6 ± 9.7
Baseline	2460.3 ± 2771.9	2460.4 ± 2327.0	1868.2 ± 1279.2
2 ms (μV)	1407.2 ± 2025.6	1255.6 ± 1272.5	803.9 ± 803.9
4 ms (μV)	1856.1 ± 2173.5	1920.8 ± 2073.9	906.9 ± 964.0
6 ms (μV)	2418.1 ± 2694.6	2821.2 ± 3104.7	1136.5 ± 962.3
8 ms (μV)	2010.8 ± 2008.6	2887.7 ± 2539.6	1053.7 ± 891.3 **
Slope	11.2 ± 293.5	242.3 ± 334.6 *	−129.6 ± 173.8 **

### Correlation analysis

**Fig. 2:**
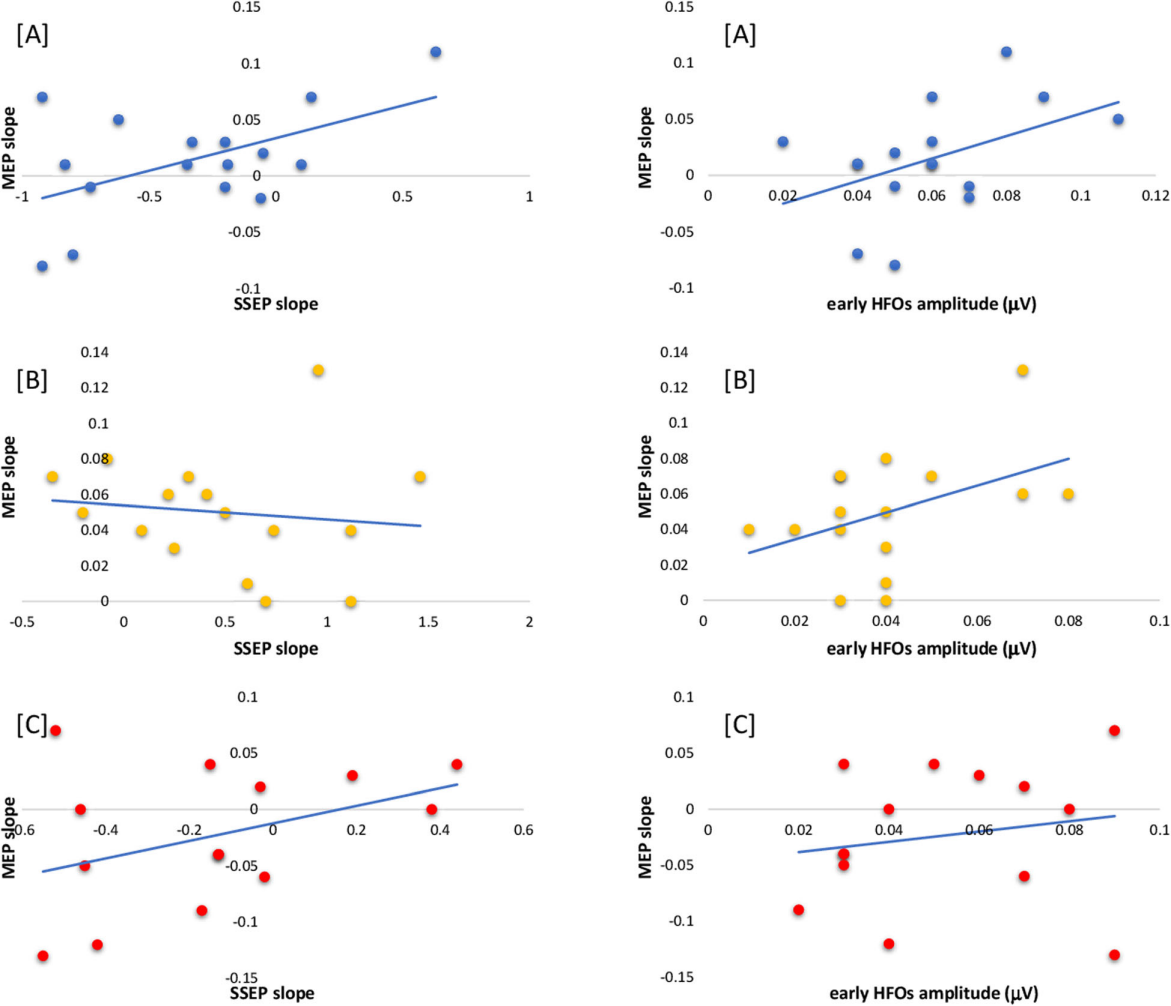
Scatter plots showing the correlation between individual motor evoked potential (MEP) slopes of the regression line between the unconditioned MEP amplitude and the 4-conditioned MEPs (y axis) and habituation of the somatosensory evoked potential (SSEP) expressed as the slope of the regression line over 3 blocks of sequential recordings (x axis - left panels) or amplitude of the early burst of SSEP HFOs (x axis – right panels) in healthy volunteers (**a**) and in migraine patients recorded between (**b**) or during (**c**) attacks

The correlation analysis showed that the slope of MEP amplitudes correlated positively with that of SSEP in HVs (*r* = 0.540, *p* = 0.031), while this correlation was not found in the two groups of patients (*r* = − 0.086, *p* = 0.751 in MO and *r* = 0.399, *p* = 0.158 in MI). Similarly, there was a significant positive correlation between the MEP slope and the amplitude of early somatosensory HFOs in HVs (*r* = 0.510, *p* = 0.044), but not in the 2 groups of patients (*r* = 0.439, *p* = 0.089 in MO and *r* = 0.208, *p* = 0.476 in MI) (Fig. 2).

No clinical variable correlated with neurophysiological results.

## Discussion

The main conclusion of this study is that the neurophysiological mechanisms underlying short-latency afferent inhibition (SAI) are dysfunctional in migraine patients: in between attacks SAI is reduced compared to healthy volunteers, while it is increased during an attack. In healthy volunteers, SAI correlates with both habituation of SSEP and amplitude of thalamocortical activity indexed by somato-sensory high frequency oscillations (HFOs). By contrast, such correlation is absent in both subgroups of migraineurs.

The mechanisms underlying SAI are complex. SAI could be induced by the somatosensory projections that reach the dorsomedial and intralaminar thalamic nuclei. In fact, in patients with a thalamic stroke, SAI is abolished, while SSEP remains intact [23]. The primary role of the thalamus in generating SAI can also be exerted indirectly via cortico-cortical modulation of the motor cortex by the somatosensory cortex, as suggested by neuromodulation studies. The latter indicate that experimental protocols raising extracellular GABA levels inhibit S1 pyramidal cells, which reduces their cortico-cortical inhibitory activity on M1 and thus reduces SAI [24]. Concordantly, benzodiazepines, GABA_A_ receptor agonists, reduce SAI [25]. Moreover, SAI is modulated by acetylcholine: it increases after administration of an acetylcholinesterase inhibitor [26] and decreases after administration of a muscarinic receptor antagonist [27]. In conditions where a central deficit of acetylcholine plays an important pathophysiological role, such as Alzheimer’s disease [26] and mild cognitive impairment [28], SAI is reduced. Taken together, these studies suggest that SAI involves a cholinergic pathway whose activity is modulated by GABA [11].

In patients recorded between attacks, SAI was reduced in our study, while it was enhanced during attacks respective to healthy volunteers. As previously shown, the maximum inhibition occurred at N20 + 2 ms in both HV and patients. The difference between ictal and interictal recordings became evident only when the median nerve stimulus was given at N20 + 8 ms before the TMS stimulus. Similar abnormalities with long ISIs, even exceeding N20 + 8 ms, were observed in various pathologies [11, 29–31], underlining the fact that short and long afferent inhibitions may be mediated by partially different cortical circuits [11]. This underscores that exploring the sensorimotor system at long ISIs between 200 to 1000 ms (long-latency afferent inhibition, LAI) would be of interest in migraine [11].

Migraine-cycle dependent changes similar to those found for SAI occurred in thalamocortical activity indexed by the amplitude of early SSEP high frequency oscillations, and in SSEP habituation. The latter findings are in accordance with our previous studies in similar groups of patients [2, 12, 13]. The difference between interictal and ictal SAI may thus be due respectively to a decrease and an increase of facilitatory thalamocortical cholinergic activity on GABAergic network activity in the motor cortex. In a previous study, in which we conditioned the MEP by an electrical stimulation of the ulnar nerve 10 msec earlier, the MEP inhibition correlated with amplitude of SSEP early HFOs in a mixed group of healthy volunteers and interictal migraineurs [16]. In the present study, we found a significant positive correlation between the MEP slope and early HFO amplitude or SSEP habituation only in healthy controls. The lack of such correlation in the two subgroups of patients could be related to a dysfunctioning thalamo-cortical control and/or to the greater variance of the thalamo-cortical drive between patients. It is of interest that the interictal reduction of SAI is reminiscent of another neurophysiological abnormality previously reported in children with migraine, i.e. the reduced inhibition of SSEP amplitude after paired stimuli [32], which may be yet another finding in favor of a less efficient subcortical inhibition of sensory cortices [33]. Taken together, the results emphasize the fundamental role of the thalamus in controlling cortical inhibitory mechanisms.

Alaydin et al. reported normal SAI in migraineurs between attacks contrasting with potentiation of the conditioned MEP amplitude in the preictal and ictal phases [10]. The discrepancy with our findings is probably due to the different experimental protocol. While we used 4 different short ISIs, adjusted to the SSEP N20 latency, Alaydin et al. used a conditioning stimulus set at 2 times the sensory threshold and a fixed predetermined stimulus interval of 21 milliseconds. After conditioning by median nerve stimulation, MEP increases instead of decreases may occur with long ISIs [19]. Compared to the Magstim Rapid device we used, the Magstim 200 used in the other study delivers more intense stimuli at the same output percentage and it is known that the SAI magnitude diminishes with increasing TMS intensities [34]. Finally, although no correlation was found with SAI, the migraine history duration was significantly longer in patients we recorded during an attack than in those who were interictal, which might have contributed to the difference in results.

Besides this heterogeneity in duration of the migraine disease between patients’ groups, our study has some additional limitations. For instance, we were not able to record the same patients during and outside the attack, which would have allowed using each patient as its own control and strengthened our conclusions. Regarding the electrophysiological protocol, averaging 5 stimuli per condition might be not enough to obtain stable SAI measure, although in previous studies reliable SAI results were obtained by averaging 3 trials only per condition [29, 30, 35–37].

## Conclusion

We have shown that short-latency afferent inhibition in the motor cortex changes with the phase of the migraine cycle. As suggested by our data, these migraine-state dependent fluctuations may be related to those of cholinergic thalamocortical activity that malfunctions in migraine. Like the lack of somatosensory habituation, the abnormal short-latency afferent inhibition supports a dysfunction of short-term cerebral plasticity mechanisms subtending the recurrence of migraine attacks.

Further studies are necessary in order to verify if these dysfunctional plastic mechanisms of the migrainous brain can be normalized by pharmacological and non-pharmacological interventions and if they are similar or different in chronic migraine with or without medication overuse. Nevertheless, since the mechanisms of SAI might be related to several cognitive domains [28, 38], it seems of interest to search in migraine for a possible relation between SAI abnormalities and the known ictal [39–42] and interictal [43–45] impairments of cognitive performance.

## Data Availability

Clinical, neurophysiological and statistical data will be available upon request from any qualified investigator.

## Abbreviations

EP: Evoked potential
HFO: High-frequency oscillation
HV: Healthy volunteer
ISI: Interstimulus interval
LAI: Long-latency afferent inhibition
MEP: Motor evoked potential
MI: Migraine without aura during the attack
MO: Migraine without aura between attacks
SAI: Short-latency afferent inhibition
SSEP: Somatosensory evoked potentials
TMS: Transcranial magnetic stimulation
